# The effects of NutraGen supplement on cattle growth performance, energetic efficiency, carcass characteristics, and characteristics of digestion in calf-fed Holstein steers

**DOI:** 10.3389/fvets.2023.1039323

**Published:** 2023-02-24

**Authors:** Pedro H. V. Carvalho, Brooke C. Latack, Marcos V. C. Ferraz Junior, Ruben Flores, Gerson Sanchez-Cruz, Martin F. Montaño, Richard A. Zinn

**Affiliations:** ^1^Department of Animal Science, University of California, Davis, Davis, CA, United States; ^2^Cooperative Extension, Division of Agriculture and Natural Resources, University of California, Holtville, Holtville, CA, United States; ^3^Departamento de Zootecnia Instituto de Ciências Sociais, Educação e Zootecnia, Universidade Federal do Amazonas, Parinitns, Brazil; ^4^Department of Nutrition and Biotechnology of Ruminants, Instituto de Investigaciones en Ciencias Veterinarias-UABC, Mexicali, Baja California, México

**Keywords:** Holstein, feedlot, performance, heat-stress, cattle

## Abstract

Evaluation of the effects of feeding NutraGen supplement (NutraGen, NTG; Phibro Animal Health, Teaneck, NJ, USA) on growth performance, energetic efficiency, carcass characteristcs, and characteristics of digestion in calf-fed Holstein steers fed a conventional growing-finishing diet. Trial 1 evaluated growth performance, dietary energetics and carcass characteristics. Two hundred Holstein steer calves (134 ± 5 kg) were blocked by initial body weight (BW) and randomly assigned to 40 pens (5 steers/pen). Dietary treatments consisted of a steam-flaked corn-based growing-finishing diet supplemented with 0, 0.2, 0.4, or 0.6% NTG (DM basis). In trial 2, four Holstein steers (170 ±6 kg) with cannulas in the rumen and proximal duodenum were used in a 4 × 4 Latin square experiment to evaluate digestibility and ruminal characteristics using the treatments from trial 1. Compared to non-supplemented cattle, NTG increased BW (2.0%, *P* = 0.02) and tended to increase ADG (3.6%, *P* = 0.07) during the initial 56 d period. However, there were no treatment effects on overall growth performance and efficiency of dietary energy utilization after the first 56 days (*P* > 0.10). Supplementation of NTG increased (linear effect; *P* ≤ 0.03) longissimus muscle area and kidney, pelvic, and heart fat. There was no effect (*P* ≥ 0.05) of NTG supplementation on other carcass characteristics, liver abscess incidence, or liver abscess scars. Supplementation decreased the molar proportion of ruminal propionate (*P* = 0.05) and tended to increase acetate:propionate molar ratio (*P* = 0.09). However, there was no effect of NTG supplementation on ruminal and total tract diet digestion. NTG increased performance of Holstein steers during the first 56 d on feed in the feedlot. In addition, the steers had an increase in KPH fat and LM area, indicating that the additive induced change in metabolism of the steers.

## Introduction

Recently, there has been an increased concern about the over-routine use of supplemental antibiotics, which has increased the investigation of potential non-antibiotic alternatives in cattle diets (1). NutraGen (NTG; Phibro Animal Health, Teaneck, NJ, USA) contains similar properties to OmniGen (OMG; Phibro Animal Health, Teaneck, NJ, USA), which is used in the dairy industry (2). Previous research has reported that the use of this blend in dairy cows' diets includes improved metabolic response to a glucose tolerance test (2), modulation of inflammatory responses (3), increased milk production (4), and enhanced immunomodulatory response to heat stress (5, 6). The latter may be particularly relevant for enhancing growth performance responses in feedlot cattle during extreme ambient temperatures. The NTG is a blend of silicon dioxide, calcium aluminosilicate, sodium aluminosilicate, dehydrated brewer's yeast, mineral oil, calcium carbonate, rice hulls, niacin, biotin, d-calcium pantothenate, choline chloride, thiamine mononitrate, pyridoxine hydrochloride, riboflavin-5-phosphate, and folic acid.

Although some of these compounds have been studied in the beef industry individually, to the authors' knowledge, the mechanism of action of NTG and OMG on heat stress is still unknown. Wang et al. (7) observed that OMG supplementation increased the expression of neutrophil associated genes in peri-parturient dairy cows, such as interleukin-1b converting enzyme, interleukin-4 receptor, interleukin-8 receptor and thymopoeitin-α (7). Feeding OMG for 2 weeks increased mammary inflammatory response and antigen presentation to mammary infections caused by a single strain of mastitis pathogens (Streptococcus uberis, Escherichia coli, and Staphylococcus aureus) in a mouse model of infection, suggesting OMG could decrease the incidence of infectious diseases in ruminants (8). These reports together indicated that OMG might reduce the incidence of infectious diseases in ruminants. However, no information has been published on the effects of supplemental NTG on cattle growth performance and digestive function when cattle are fed a common steam-flaked corn-based diet.

Moreover, there is no information on the potential benefits of feeding NTG as a heat load mitigator for calf-fed Holstein steers in the southern desert region of the United States. A region characterized by its extreme heat conditions during the summer months. Therefore, the objective of the current study was to evaluate the effects of NTG supplementation on growth performance, energetic efficiency, carcass characteristics, and digestive function in calf-fed Holstein steers.

## Materials and methods

Procedures for animal care and management were conducted under a protocol (#20548) approved by the University of California, Animal Use and Care Advisory Committee.

### Trial 1

#### Cattle management and treatments

Two hundred Holstein steer calves (130.9 ± 4 kg) were utilized to evaluate the influence of NTG supplementation on growth performance, dietary energetics, and carcass characteristics. The trial was initiated on January 14, 2020, and completed following a 322-d feeding period (December 1, 2020). Calves were purchased from a commercial calf ranch (CalfTech, Tulare, CA). Upon arrival at the University of California Desert Research and Extension Center (Holtville, CA), calves were vaccinated for IBR, BVD, PI3, and BRSV (Bovi-shield^®^ Gold One Shot, Zoetis Animal Health, New York, NY), clostridials (Ultrabac^®^ 7, Zoetis Animal Health, New York, NY), treated against internal and external parasites (Dectomax, Zoetis Animal Health, New York, NY), injected with 1,500 IU vitamin E (as d-alpha-tocopherol) 500,000 IU vitamin A (as retinyl-palmitate) and 50,000 I.U. vitamin D3 (Vital E-AD, Stuart Products, Bedford, TX), and 300 mg tulathromycin (Draxxin, Zoetis Animal Health, New York, NY). Calves were blocked by initial shrunk (off truck) weight and randomly assigned within weight groupings to 40 pens (5 steers/pen, 10 pens/treatment). On day 28, calves received the Ultrabac^®^ 7 booster vaccination and were vaccinated with Endovac-Beef (Endovac Animal Health, Columbia, MO). On d 56 calves received the Endovac-Beef booster vaccination. Pens were 62 m^2^ with 25 m^2^ overhead shade, automatic waterers, and 2.4 m fence-line feed bunks. Steers were allowed *ad libitum* access to feed and water. Fresh feed was provided daily.

Dietary treatments are shown in Table 1, consisting of a steam-flaked corn-based diet supplemented with (DM basis): (1) no feed additive; (2) 0.2% of NTG; (3) 0.4% of NTG; (4) 0.6% of NTG. Nutragen doses were based on a previous study on dairy cows, and no feed additive was included in the diet. Diets were prepared weekly and stored in plywood boxes in front of each pen. On d 112 and 224, all steers were reinjected subcutaneously with 500,000 IU vitamin A (Vital E-A + D, Stuart Products, Bedford, TX) and implanted with Revalor-S (Intervet, Millsboro, DE). The health status of cattle was checked daily. Cattle were monitored daily by trained personnel for signs of illness or pinkeye. Cattle with signs of illness were pulled out, classified as morbid, and treated with an antimicrobial if the rectal temperature was ≥ 39.5°C. Antimicrobial treatments were conducted following a veterinarian's recommendation. A post-treatment interval of 3 days was implemented after the first and second treatments. If cattle remained morbid after the third treatment and the prognosis of a full recovery was unlikely, cattle were removed from the study (two animals died during the experiment for causes unrelated to dietary treatments).

**Table 1:** Composition of experimental diets (DM basis).

	**NutraGen** ^*****b*****^ **level, %**			
**Item**	**0**	**0.2**	**0.4**	**0.6**
**Ingredient composition, % DM**				
Sudangrass hay	8.00	8.00	8.00	8.00
Alfalfa hay	4.00	4.00	4.00	4.00
Tallow	2.50	2.50	2.50	2.50
Molasses, cane	4.00	4.00	4.00	4.00
Distillers Grains w/solubles	10.00	10.00	10.00	10.00
Steam flaked corn	68.12	67.92	67.72	67.52
Urea	1.15	1.15	1.15	1.15
Limestone	1.68	1.68	1.68	1.68
Dicalcium phosphate	0.10	0.10	0.10	0.10
Magnesium oxide	0.15	0.15	0.15	0.15
TM Salt^a^	0.30	0.30	0.30	0.30
NutraGen^b^	0.00	0.20	0.40	0.60
**Nutrient composition, DM basis** ^c^				
Dry matter, %	87.9	87.9	87.9	87.9
NEm, Mcal/kg	2.21	2.20	2.20	2.20
NEg, Mcal/kg	1.53	1.52	1.52	1.52
Crude protein, %	14.3	14.3	14.3	14.3
Rumen DIP, %	62.7	62.7	62.7	62.7
Rumen UIP, %	37.3	37.3	37.3	37.3
Ether extract, %	6.70	6.70	6.70	6.70
Ash, %	5.76	5.76	5.76	5.76
Nonstructural CHO, %	58.0	58.0	58.0	58.0
NDF, %	17.7	17.7	17.7	17.7
Calcium, %	0.80	0.80	0.80	0.80
Phosphorus, %	0.35	0.35	0.35	0.35
Potassium, %	0.77	0.77	0.77	0.77
Magnesium, %	0.28	0.28	0.28	0.28
Sulfur, %	0.19	0.19	0.19	0.19

^a^Trace mineral salt contained: CoSO4, 0.068%; CuSO4, 1.04%; FeSO4, 3.57%; ZnO, 0.75%; MnSO4, 1.07%; KI, 0.052%; and NaCl, 93.4%.

^b^NutraGen (Phibro Animal Health, Ridgefield Park, NJ) is a proprietary formulation containing a mixture of silicon dioxide, calcium aluminosilicate, sodium aluminosilicate, dehydrated brewers yeast, mineral oil, calcium carbonate, rice hulls, niacin, biotin, d-calcium pantothenate, choline chloride, thiamine mononitrate, pyridoxine hydrochloride, riboflavin-5-phosphate and folic acid.

^b^Based on tabular values for individual feed ingredients (33).

Steer full body weight (BW) was recorded every 28 days until the end of the experiment to monitor live weight changes. Steers were not denied feed or water before weighing. In determining ADG, interim and final weights were reduced by 4% to account for digestive tract fill (9). From March 9, 2020, to the end of the experiment, SmaX-tec intraruminal boluses (SmaX-tec animal care technology^®^, Graz, Austria) were orally inserted into the rumen (1 steer per pen) to monitor the ruminal temperature. Continuous real-time temperature data was retrieved using a monitoring device located in the proximity of cattle pens.

#### Carcass measurements

Hot carcass weights (HCW) and liver abscess incidence [based on size and number, scaled as 0, A–, A, and A+; (10)], as well as liver scarring measures, were obtained at the time of slaughter. After carcasses were chilled for 24 h, the following measurements were obtained: Longissimus muscle (LM) area (cm^2^) by direct grid reading of the muscle at the 12th rib; subcutaneous fat (cm) over the LM at the 12th rib taken at a location 3/4 the lateral length from the chine bone end (adjusted for unusual fat distribution); kidney, pelvic and heart fat (KPH) as a percentage of HCW; marbling score [(11); using 3.0 as minimum slight, 4.0 as minimum small, 5.0 as minimum modest, 6.0 as minimum moderate, etc.], and preliminary as well as estimated retail yield of boneless, closely trimmed retail cuts from the round, loin, rib and chuck as a percentage of HCW [Yield, % = 52.56–1.95 × subcutaneous fat−1.06 × KPH + 0.106 × LM area−0.018 × HCW; (12)].

#### Estimation of dietary net energy

Daily energy gain (EG; Mcal/d) was calculated by the equation: EG = ADG^1.097^ 0.0557W^0.75^, where W is the mean shrunk B.W. [kg; (13)] Maintenance energy (EM) was calculated by the equation: EM = 0.086W ^0.75^. Dietary NEg was derived from NEm by the equation: NEg = 0.877 NEm−0.41 (14). Dry matter intake is related to energy requirements and dietary NEm according to the equation: DMI = (EM/NEm) + (EG/(0.877NEm−0.41). From this relationship, dietary NE can be resolved by means of the quadratic formula: x = (–b – √ b2 – 4ac)/ 2c, where: *x* = NEm, a = – 0.42 EM, b = 0.887 EM + 0.41 DMI + EG, and c = – 0.887 DMI (15).

#### Weather measurement and temperature and humidity index estimation

Climatic variables (ambient temperature and relative humidity) were obtained every hour from an on-site weather station (California Irrigation Management Information System; Meloland Station) throughout the experimental period. The temperature humidity index was calculated using the following formula THI = (0.8 × Ta) + [(H/100) × (Ta – 14.4)] + 46.4, where Ta is air temperature (°C) and H is relative humidity (16, 17); Min = minimum; Max = maximum.

#### Statistical design and analysis

Pens were used as experimental units. The experimental data were analyzed as a randomized complete design experiment according to the following statistical model: Yij = μ + Bi + Tj + Eij, where μ is the common experimental effect, Bi represents the initial weight group effect (df = 6), Tj represents the dietary treatment effect (df = 3), and Eij represents the residual error (df = 18). Treatment effects were tested using the following contrasts: 0 vs. NTG and linear and quadratic polynomials to assess the effect of dosage (Stastix 10, Analytical Software, Tallahassee, FL).

### Trial 2

#### Cattle management and treatments

Four Holstein steers (170 ±6 kg) with cannulas in the rumen and proximal duodenum were used in a 4 × 4 Latin square experiment. Treatments are the same as in Trial 1 (Table 1). A single basal diet was prepared with 0.3% chromic oxide as a digesta marker. Corresponding amounts of NTG (0, 0.2, 0.4, and 0.6% of diet DM) were top-dressed on the basal diet at the time of feeding. Dry matter intake was restricted to 2.2% of live weight to avoid feed refusals. Diets were fed at 0,800 and 2,000 daily. Experimental periods consisted of a 17-d diet adjustment period followed by a 4-d collection period. During the collection period, duodenal and fecal samples were obtained from all steers twice daily: d 1, 0750, and 1,350; d 2, 0,900, and 1,500; d 3, 1,050, and 1,650; and d 4, 1,200 and 1,800. Individual samples consisted of approximately 700 ml duodenal chyme and 200 g (wet basis) fecal material. Additionally, on d4 of the collection period at 1,200 h, 100 mL of ruminal fluid was obtained from each steer *via* the ruminal cannula. Ruminal fluid pH was determined on freshly collected samples. Ruminal fluid was then strained through 4 layers of cheesecloth. Freshly prepared 25% (wt/vol) *m*-phosphoric acid (2 mL) was added to 8 mL of the strained ruminal fluid, centrifuged (17,000 × g for 10 min) and supernatant fluid stored at −20° C for VFA analysis [direct injection gas chromatography, using HP 5890A gas chromatograph, Hewlett Packard, Palo Alto, CA; DB-FFAP column, J&W Instruments, New Brighton, MN; WSFA-2 VFA standards, Supelco Analytical, Bellefonte, PA; 3-methyvaleric acid internal standard, TCI America, Portland, OR; (18)]. Duodenal and fecal samples from each steer within each collection period were composited for analysis. Upon completion of the experiment, ruminal fluid was obtained *via* the ruminal cannula from all steers and composited for the isolation of ruminal bacteria by differential centrifugation (19). Feed, duodenal fluid, and fecal samples were subjected to the following analysis: DM [oven drying at 105°C until no further weight loss; method 930.15; (20)]; ash [method 942.05; (20)], Kjeldahl N [method 984.13; (20)]; aNDFom (21), corrected for NDF-ash, incorporating heat stable α-amylase (Ankom FAA, Ankom Technology, Macedon, NY) at 1 mL per 100 mL of NDF solution]; chromic oxide (22); and starch (23). Duodenal samples were also analyzed for ammonia N (method 941.04, 20) and purines (24). Duodenal flow and fecal excretion of DM were determined based on marker ratio using chromic oxide. Microbial organic matter (MOM) and nitrogen (MN) leaving the abomasum were estimated using purines as microbial markers (24). Organic matter (OM) fermented in the rumen is considered equal to OM intake minus the difference between the amount of total OM reaching the duodenum and MOM reaching the duodenum. Feed N escape to the small intestine is considered equal to total N leaving the abomasum minus ammonia-N, MN, and endogenous N [0.195 × BW^0.75^; (25)]. Methane production was estimated based on the theoretical fermentation balance for observed molar distribution of VFA and OM fermented in the rumen (26).

#### Statistical design and analysis

The trial was analyzed as a balanced 4 × 4 Latin square according to the following statistical model: *Y*_*ijk*_ = μ+ *A*_*i*_ + *P*_*j*_ + *T*_*k*_ + *E*_*ijk*_, where *Y*_*ijk*_ is the response variable, μ is the common experimental effect, *A*_*i*_ is the steer effect, *P*_*j*_ is the period effect, *T*_*k*_ is the treatment effect, and *E*_*ijk*_ is the residual error. Treatment main effects were assessed by means of orthogonal polynomials.

## Results

In the present study, NTG was supplemented at 0, 0.2, 0.4, and 0.6% of diet DM. During the first 56 days of the study, steers consumed 0, 59, 119, and 178 mg per kg of live BW per day, respectively. However, the observed intake from 112 to the end of the experiment averaged 0, 39, 79, and 117 mg per kg of live BW per day, respectively. Treatment effects on health, growth performance, and estimated dietary NE are shown in Table 2. Morbidity and mortality were low and not affected (*P* > 0.20) by dietary treatments, averaging 5.5 and 0.5%, respectively. Supplementation of NTG increased (quadratic effect; *P* = 0.05) shrunk live weight on d 56 of the trial, with cattle supplemented with 0.2% NTG being the heaviest. Moreover, NTG supplementation tended to increase (*P* = 0.07) ADG from d 1–56 and decrease (*P* = 0.06) ADG from d 112–322. There was no effect of NTG on growth at any other time and no effect on DMI, feed efficiency, or efficiency of energy utilization (*P* > 0.10) (Table 2).

**Table 2:** Effects of feeding NutraGen supplement on health, growth performance and dietary net energy utilization.

	**NutraGen**^**a**^ **(%)**					***P*** **-value**		
**Item**	**0**	**0.2**	**0.4**	**0.6**	**SEM**	**L**	**Q**	**0 vs. TMT**
Days on test	322	322	322	322				
Pen replicated	10	10	10	10				
Morbidity, %	6.0	10.0	4.0	2.0	3.1	0.20	0.34	0.85
Mortality, %	0.0	2.0	0.0	0.0	1.0	0.66	0.33	0.57
**Live weight**^2^ **(kg)**								
Initial	129.8	132.3	130.5	130.9	0.71	0.63	0.16	0.10
56 d	196.3	202.0	199.3	199.3	1.4	0.31	0.05	0.02
112 d	281.9	284.8	283.8	283.5	1.9	0.69	0.42	0.37
Final	586.2	587.1	581.4	590.6	5.2	0.75	0.43	0.97
**ADG (kg)**								
1–56 d	1.19	1.25	1.23	1.22	0.02	0.36	0.12	0.07
56–112 d	1.53	1.48	1.51	1.50	0.02	0.64	0.36	0.25
1–112 d	1.36	1.36	1.37	1.36	0.02	0.83	0.79	0.78
112–322 d	1.45	1.44	1.42	1.46	0.02	0.84	0.23	0.06
1–322 d	1.42	1.41	1.40	1.43	0.02	0.79	0.32	0.84
**DMI (kg/d)**								
1–56 d	4.82	4.96	4.90	4.91	0.07	0.49	0.35	0.20
56–112 d	6.99	7.01	7.03	7.02	0.09	0.85	0.89	0.85
1–112 d	5.91	5.98	5.97	5.96	0.07	0.65	0.59	0.46
112–322 d	8.65	8.45	8.51	8.56	0.11	0.68	0.26	0.27
1–322 d	7.69	7.59	7.62	7.66	0.09	0.84	0.45	0.50
**ADG/DMI (kg/kg)**								
1–56 d	0.246	0.252	0.251	0.249	0.003	0.64	0.30	0.30
56–112 d	0.219	0.211	0.215	0.214	0.003	0.51	0.27	0.16
1–112 d	0.230	0.228	0.230	0.229	0.003	0.85	0.87	0.72
112–322 d	0.168	0.170	0.167	0.171	0.002	0.55	0.77	0.52
1–322 d	0.184	0.186	0.184	0.187	0.002	0.62	0.84	0.59
**Dietary ME (Mcal/kg)**								
**Maintenance**								
1–112 d	1.88	1.88	1.88	1.88	0.02	0.89	0.90	0.96
112–322 d	2.27	2.31	2.27	2.31	0.03	0.47	0.96	0.33
1–322 d	2.19	2.22	2.18	2.22	0.02	0.58	0.91	0.48
**Gain**								
1–112 d	1.24	1.24	1.24	1.24	0.01	0.89	0.90	0.96
112–322 d	1.58	1.62	1.58	1.62	0.02	0.47	0.96	0.33
1–322 d	1.51	1.53	1.50	1.53	0.02	0.58	0.91	0.48
**Observed/ expected dietary NE**								
**Maintenance**								
1–112 d	0.85	0.85	0.85	0.85	0.01	0.89	0.90	0.96
112–322 d	1.03	1.05	1.03	1.05	0.01	0.47	0.96	0.33
1–322 d	0.99	1.00	0.99	1.00	0.01	0.58	0.91	0.48
**Gain**								
1–112 d	0.81	0.81	0.81	0.81	0.01	0.89	0.90	0.96
112–322 d	1.03	1.06	1.03	1.06	0.01	0.47	0.96	0.33
1–322 d	0.99	1.00	0.98	1.00	0.01	0.58	0.91	0.48

^1^NutraGen (Phibro Animal Health, Ridgefield Park, NJ) is a proprietary formulation containing a mixture of silicon dioxide, calcium aluminosilicate, sodium aluminosilicate, dehydrated brewers yeast, mineral oil, calcium carbonate, rice hulls, niacin, biotin, d-calcium pantothenate, choline chloride, thiamine mononitrate, pyridoxine hydrochloride, riboflavin-5-phosphate and folic acid.

^2^Live weight reduced by 4% to account for gut fill.

Treatment effects on carcass characteristics, liver abscess incidence, liver scars measures, and morbidity are shown in Table 3. Dietary supplementation of NTG increased LM area (linear effect; *P* = 0.03) and KPH fat (linear effect; *P* = 0.01). However, there was no effect (*P* ≥ 0.12) of NTG supplementation on other carcass characteristics, liver abscess incidence, or liver abscess scars.

**Table 3:** Effects of feeding NutraGen supplement on carcass characteristics, liver abscess, and cattle morbidity.

	**NutraGen**^**a**^ **(%)**					***P*** **-value**		
**Item**	**0**	**0.2**	**0.4**	**0.6**	**SEM**	**L**	**Q**	**0 vs. TMT**
Carcass weight (kg)	364.0	364.6	361.1	366.8	3.2	0.75	0.43	0.97
Dressing percentage (%)	62.0	62.3	61.9	62.2	0.22	0.81	0.93	0.60
KPH fat^b^ (%)	3.10	3.14	3.20	3.31	0.06	0.01	0.53	0.09
Fat thickness (cm)	0.83	0.79	0.73	0.78	0.04	0.32	0.29	0.24
LM area (cm^b^)	83.8	86.5	85.5	88.1	1.1	0.03	0.94	0.04
Marbling score^c^	4.35	4.32	4.53	4.41	0.10	0.41	0.71	0.59
Retail yield	51.3	51.7	51.6	51.7	0.17	0.17	0.50	0.12
Yield Grade	2.82	2.66	2.64	2.63	0.09	0.13	0.39	0.09
Abscessed liver (%)	12.0	14.0	6.0	10.0	5.0	0.54	0.84	0.73
Liver abscess scars (%)	30.0	22.0	22.0	32.0	6.2	0.83	0.16	0.52
Morbidity (%)	6.0	10.0	4.0	2.0	3.1	0.20	0.34	0.85

^a^NutraGen (Phibro Animal Health, Ridgefield Park, NJ) is a proprietary formulation containing a mixture of silicon dioxide, calcium aluminosilicate, sodium aluminosilicate, dehydrated brewers yeast, mineral oil, calcium carbonate, rice hulls, niacin, biotin, d-calcium pantothenate, choline chloride, thiamine mononitrate, pyridoxine hydrochloride, riboflavin-5-phosphate and folic acid.

^b^KPH fat as a percentage of carcass weight.

^c^Coded: minimum slight = 3.0, minimum small = 4.0, minimum modest = 5.0, minimum moderate = 6.0, and so on.

**Figure 1:**
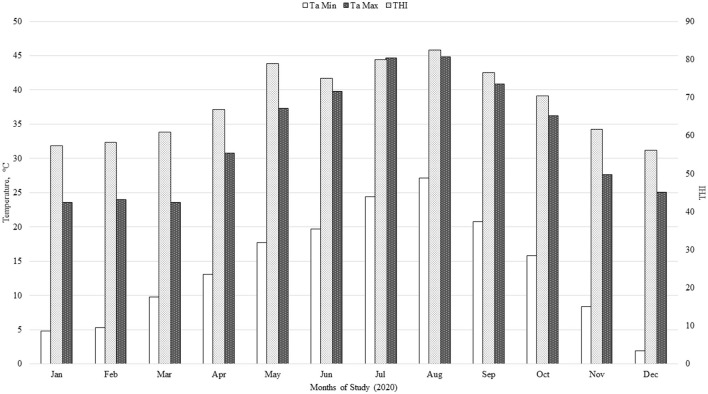
Temperature-humidity index (THI) during the 286-d feeding period: THI = (0.8 × Ta) + [(H/100) × (Ta – 14.4)] + 46.4, where Ta is air temperature (°C), and H is relative humidity (16, 17); Min = minimum; Max = maximum.

Treatment effects on minimum, average, and maximum ruminal temperature during the summer months (June, July, August, and September) are presented in Table 4. There was no effect (*P* ≥ 0.18) of NTG supplementation on minimum, average, or maximum ruminal temperature during summer months when cattle were under high ambient temperature conditions (Figure 1).

**Table 4:** Effects of feeding NutraGen supplement on ruminal temperature during summer months.

	**NutraGen**^**a**^ **(%)**					***P*** **-value**	
**Item**	**0**	**0.2**	**0.4**	**0.6**	**SEM**	**L**	**Q**
**June ruminal temp**, °**C**							
Min temp	39.3	39.2	39.3	39.3	0.05	0.92	0.53
Ave temp	40.0	39.9	40.1	39.9	0.06	0.93	0.95
Max temp	40.7	40.5	40.8	40.6	0.07	0.95	0.92
**July ruminal temp**, °**C**							
Min temp	39.4	39.3	39.4	39.4	0.07	0.49	0.57
Ave temp	40.3	40.1	40.4	40.2	0.07	0.74	0.89
Max temp	40.9	40.9	41.2	40.9	0.08	0.64	0.59
**August ruminal temp**, °**C**							
Min temp	39.5	39.4	39.5	39.5	0.06	0.77	0.45
Ave temp	40.4	40.2	40.4	40.3	0.06	0.80	0.87
Max temp	41.1	40.9	41.2	41.0	0.07	0.99	0.77
**September ruminal temp**, °**C**							
Min temp	38.9	38.9	39.1	39.0	0.05	0.18	0.66
Ave temp	39.8	39.8	39.9	39.8	0.07	0.41	0.61
Max temp	40.4	40.4	40.6	40.5	0.09	0.35	0.60

^a^NutraGen (Phibro Animal Health, Ridgefield Park, NJ) is a proprietary formulation containing a mixture of silicon dioxide, calcium aluminosilicate, sodium aluminosilicate, dehydrated brewers yeast, mineral oil, calcium carbonate, rice hulls, niacin, biotin, d-calcium pantothenate, choline chloride, thiamine mononitrate, pyridoxine hydrochloride, riboflavin-5-phosphate and folic acid.

Treatment effects on ruminal and total tract digestion and ruminal pH and VFA are shown in Tables 5, 6, respectively. There was no effect (*P* ≥ 0.12) of NTG supplementation on ruminal and total tract digestion. The supplementation of NTG decreased (*P* = 0.05) propionate concentration in the rumen by 16.5% compared to the non-supplemented diet; therefore, the ruminal acetate/propionate ratio tended (*P* = 0.09) to increase linearly with NTG supplementation. There was a quadratic effect (*P* < 0.01) on isobutyrate concentration in the rumen, with isobutyrate minimal concentration being observed when cattle were supplemented with 0.2% of NTG in the diet. Moreover, cattle supplemented with NTG had a linear increase (*P* = 0.05) in the methane/mol glucose ratio compared to non-supplemented cattle. There was no effect (*P* ≥ 0.10) of NTG supplementation on ruminal pH and other VFA concentrations.

**Table 5:** Effects of feeding NutraGen supplement on characteristics of ruminal and total tract digestion.

	**NutraGen**^**a**^ **(%)**					***P*** **-value**		
**Item**	**0**	**0.2**	**0.4**	**0.6**	**SEM**	**L**	**Q**	**0 vs. TMT**
Steer replication	4	4	4	4				
**Intake, g/d**								
DM	3,672	3,672	3,672	3,672				
OM	3,464	3,464	3,464	3,464				
NDF	554.2	554.2	554.2	554.2				
N	80.4	80.4	80.4	80.4				
Starch	1,706	1,706	1,706	1,706				
**Flow to the duodenum, g/d**								
OM	1,737	1,711	1,708	1,680	40.4	0.38	0.98	0.46
NDF	269.0	238.6	283.3	255.9	22.6	0.96	0.94	0.72
Starch	268.7	272.6	265.6	216.0	30.5	0.27	0.41	0.64
Microbial N	45.0	45.6	44.0	49.1	3.3	0.49	0.52	0.76
Nonammonia N	84.6	84.7	83.9	85.4	1.1	0.73	0.56	0.94
Feed N	30.4	29.9	30.7	27.1	2.7	0.48	0.58	0.73
**Ruminal**								
OM	62.9	63.8	63.4	65.7	0.02	0.34	0.71	0.50
NDF	51.5	57.0	48.9	53.8	0.04	0.96	0.94	0.72
Starch	84.2	84.0	84.4	87.3	0.02	0.27	0.41	0.64
Feed N	62.2	62.8	61.8	66.3	0.03	0.48	0.58	0.73
Microbial efficiency^2^	20.7	20.5	20.0	21.6	1.2	0.69	0.48	0.99
Protein efficiency^3^	1.05	1.05	1.04	1.06	0.01	0.73	0.56	0.94
**Fecal excretion, g/d**								
DM	803.2	784.6	796.9	805.7	22.4	0.85	0.57	0.78
OM	672.1	656.4	655.3	662.1	23.9	0.78	0.65	0.63
NDF	275.5	265.2	254.7	263.0	14.2	0.50	0.52	0.42
Starch	10.6	10.5	8.49	8.46	1.8	0.34	0.99	0.51
N	23.4	22.8	23.2	23.5	0.61	0.82	0.51	0.74
**Total tract digestion, %**								
DM	78.1	78.6	78.3	78.1	0.61	0.85	0.57	0.78
OM	80.6	81.1	81.1	80.9	0.01	0.78	0.65	0.62
NDF	50.3	52.2	54.0	54.2	0.03	0.50	0.52	0.42
Starch	99.4	99.4	99.5	99.5	0.001	0.34	0.99	0.51
N	70.9	71.7	71.1	70.8	0.01	0.82	0.51	0.74

^a^NutraGen (Phibro Animal Health, Ridgefield Park, NJ) is a proprietary formulation containing a mixture of silicon dioxide, calcium aluminosilicate, sodium aluminosilicate, dehydrated brewers yeast, mineral oil, calcium carbonate, rice hulls, niacin, biotin, d-calcium pantothenate, choline chloride, thiamine mononitrate, pyridoxine hydrochloride, riboflavin-5-phosphate and folic acid.

^b^Duodenal microbial N, g kg^−1^ OM fermented in the rumen.

^c^Duodenal non-ammonia N, g g^−1^ N intake.

**Table 6:** Effects of feeding NutraGen supplement on ruminal pH and VFA concentrations.

	**NutraGen**^**a**^ **(%)**					***P*** **-value**		
**Item**	**0**	**0.2**	**0.4**	**0.6**	**SEM**	**L**	**Q**	**0 vs. TMT**
Ruminal pH	5.91	5.95	6.06	6.10	0.12	0.26	0.98	0.41
Total VFA, mM	72.1	74.4	71.9	75.9	8.6	0.82	0.92	0.85
**Ruminal VFA, mol/100 mol**								
Acetate	49.4	48.9	51.3	52.3	2.0	0.26	0.72	0.55
Propionate	32.5	28.1	27.2	26.1	1.9	0.06	0.43	0.05
Isobutyrate	0.69	0.38	0.45	1.01	0.11	0.08	< 0.01	0.54
Butyrate	11.9	13.9	14.3	14.9	1.2	0.13	0.58	0.12
Isovalerate	1.62	2.10	2.32	1.95	0.32	0.42	0.23	0.22
Valerate	3.90	6.56	4.39	3.63	1.9	0.75	0.42	0.69
Acetate/propionate	1.59	1.88	1.98	2.04	0.16	0.09	0.52	0.09
Methane/mol glucose^b^	0.43	0.46	0.48	0.50	0.02	0.05	0.71	0.07

^a^NutraGen (Phibro Animal Health, Ridgefield Park, NJ) is a proprietary formulation containing a mixture of silicon dioxide, calcium aluminosilicate, sodium aluminosilicate, dehydrated brewers yeast, mineral oil, calcium carbonate, rice hulls, niacin, biotin, d-calcium pantothenate, choline chloride, thiamine mononitrate, pyridoxine hydrochloride, riboflavin-5-phosphate and folic acid.

^b^Methane, mol/mol glucose equivalent fermented.

## Discussion

Although this product had not been previously tested in cattle under the conditions of the present study, we hypothesized that NTG supplementation might enhance growth performance, dry matter intake, and apparent digestibility of nutrients, ruminal parameters, and carcass characteristics of calf-fed Holstein steers. According to the manufacturer, NTG combines natural components with immunostimulant function, especially under stressful circumstances. Previous research reported that most respiratory diseases in the feedlot occurred within the first 30 days after arrival (27). Therefore, the greater growth performance observed in calves supplemented with NTG during the first weeks of the experiment may be associated with a greater immune response to challenges being faced during the receiving period, as has been suggested in previous studies (1–4, 6). However, in agreement with the current study, Colombo et al. (6) did not observe an overall effect on growth performance when crossbred yearling cattle were fed a similar immunomodulatory supplement in the feedlot. Sanchez et al. (2) observed an increase in the final BW of beef heifers supplemented with OMG, a similar product used in the current experiment. The positive effects of OMG (mainly in dairy studies; 2–4, 6) have been attributed to the enhanced immune system, growth performance responses to OMG may largely depend on the stress conditions that animals are experiencing. For comparison, no prior studies evaluate growth performance responses to NTG supplementation of calf-fed Holstein steers.

In the present study, the dosage of NTG was supplied in the percentage of DMI (0, 0.2, 0.4, and 0.6% of DM), representing an increase in the dosage consumed according to BW. However, the intake in the first 56 days (0, 59, 119, and 178 mg of NTG per kg of live BW per day) was greater compared from 112 to the end of the experiment (0, 39, 79, and 117 mg of NTG per kg of live BW per day). However, the effect of NTG on growth performance at the beginning of the experiment was not dose-dependent. Sanchez et al. (2) and Moriel et al. (28) reported an enhancement in the metabolism of beef cattle supplemented with 100 and 88 mg of OMG per kg of BW, respectively.

Brown-Brandl et al. (29) stated that under ambient conditions where THI is ≥ 78, cattle are experiencing heat stress and “danger” conditions. In the present study, cattle were experiencing THI greater than 70 for almost half of their time on feed. Colombo et al. (6) fed 111 mg of OMG per kg of live BW per day and observed that this supplementation ameliorated hyperthermia in finishing beef cattle exposed to heat stress conditions. Although supplemental OMG has been shown to decrease the negative effect of heat-stress conditions on the performance of heifers and steers in feedlot and pasture (2, 6, 28), these extreme heat conditions faced by steers in the present study may have reduced the potential benefits of feeding an immunomodulatory supplement (NTG). The weather conditions that calf-fed Holstein steers were exposed to in this study were unfavorable from a growth performance standpoint (Figure 1). Nevertheless, no death loss occurred during the period of elevated THI. Historically, very low or no mortality during the early growing phase of calf-fed Holstein has been observed at the Research Center used in this study.

The percentage of KPH fat and LM area increased linearly with increasing levels of NTG supplementation, indicating that the metabolic effect that effects of NTG on carcass characteristics were dose-dependent. The acetate and glucose are used in ruminant metabolism as substrates for fatty acid synthesis. Sanchez et al. (2) observed that heifers supplemented with OMG were more sensitive to changes in blood glucose. Moreover, Buntyn et al. (30) observed an increase in blood glucose in steers fed OMG without affecting the growth performance of feedlot steers or carcass characteristics. These findings indicate that OMG (or NTG) supplementation may affect the storage and redistribution of energy deposits: expressed by changes in KPH fat and LM area. However, research is needed to provide more information on how NTG supplementation may have brought about the observed changes.

Very little information is available that evaluates the effects of NTG on ruminal parameters digestibility. Prior research in dairy cows supplemented with OMG reported no effects of supplementation on ruminal pH and VFA concentrations (31). In the current study, NTG decreased ruminal propionate molar proportion, leading to increased estimated methane energy loss. According to these results appears that NTG may affect microorganisms that enhance methanogens. Nevertheless, components of NTG, such as dehydrated brewer's yeast, have been observed to increase ruminal propionate molar concentration in dairy cattle (32). However, as previously stated, to the authors' knowledge, there are no prior studies evaluating the effects of NTG (or OMG) on diet digestibility or ruminal kinetics in cattle fed a grain-based feedlot finishing diet.

## Conclusion

Supplementing calf-fed Holstein steers with NTG increased the live weight of steers during the initial 56 days on feed. However, despite decreasing propionate concentration, treatment effects were not appreciable on overall cattle growth performance. Calf-fed Holstein steers supplemented with NTG had a greater percentage of KPH fat and LM area. However, NTG supplementation had no major effect on diet digestibility and ruminal temperature.

## Data availability statement

The raw data supporting the conclusions of this article will be made available by the authors, without undue reservation.

## Ethics statement

The animal study was reviewed and approved by Procedures for animal care and management were conducted under protocol (#20548) approved by the University of California, Animal Use and Care Advisory Committee.

## Author contributions

All authors listed have made a substantial, direct, and intellectual contribution to the work and approved it for publication.
